# Self-Assembly of a Therapeutic Peptide Surfactant: A Small-Angle X‑ray Scattering Study

**DOI:** 10.1021/acs.langmuir.5c06529

**Published:** 2026-02-20

**Authors:** Ellen Brunzell, Kalle Sigfridsson, L. Magnus Bergström

**Affiliations:** † Department of Medicinal Chemistry, Pharmaceutical Physical Chemistry, Uppsala University, 751 23 Uppsala, Sweden; ‡ Advanced Drug Delivery, Pharmaceutical Science, R&D, AstraZeneca, 431 83 Gothenburg, Sweden

## Abstract

Amphiphilic compounds are important in many fields including pharmaceutical processes and development. Synthetic surfactants are often toxic to biological systems and frequently display poor biodegradability. Biosurfactants, such as lipopeptides and bile salts, on the other hand, can offer superior properties with regard to toxicity, biodegradability, and efficiency. Lipidated peptides are also gaining interest as therapeutic agents, as they can offer enhanced pharmacokinetic properties, compared with native peptides. The amphiphilic nature of lipidated peptides suggests that they may self-assemble into micellar structures, which can influence formulation stability and biological performance. Understanding the aggregation behavior of lipidated peptides is thus important for identifying and avoiding stability issues that could affect drug efficacy and safety. Structural characterization of self-assembled aggregates provides insight into aggregation mechanisms, which is valuable for identifying potential challenges during the production, storage, and administration of pharmaceutical peptides. By using small-angle X-ray scattering (SAXS), we have investigated the size and morphology of aggregates formed by MEDI7219, a bis-lipidated glucagon-like peptide-1 (GLP-1) analogue, in various aqueous solutions. We demonstrate that the lipopeptide MEDI7219 behaves as a surfactant with high spontaneous curvature that forms small micelles with a clear core-and-shell structure in aqueous solvents. The aggregation numbers of the micelles vary in the range of 5–8 and are found to be surprisingly insensitive to environmental conditions such as type of electrolyte and tonicity modifier, different buffers, and temperature, while exhibiting very low critical micelle concentrations (cmc).

## Graphical abstract

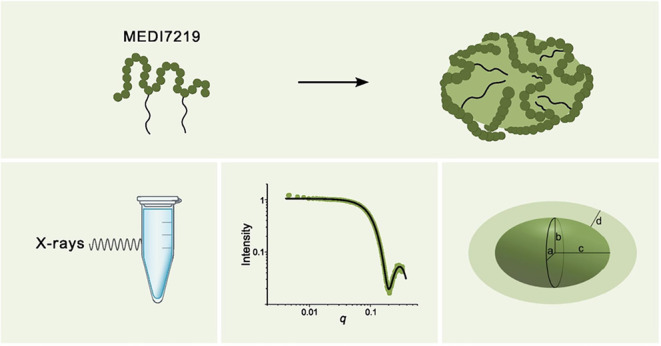

## Introduction

Amphiphilic molecules that self-assemble into micelles on their own in aqueous solvents above a certain, more or less, well-defined concentration are usually referred to as surfactants. They belong to an important type of compound with applications in several fields and industrial processes such as cleaning, food industry, and pharmaceutics.^1^ The wide use of surfactants is making biosurfactants a rising research topic, for instance, as a way to find more environmentally friendly alternatives to conventional surfactants. Synthetic surfactants are often derived from mineral oils, are toxic to many organisms, and usually have poor biodegradability.^2^ Surface-active compounds with biological origin, such as biosurfactants produced in microorganisms, bile salts, or peptide amphiphiles, are more readily biodegradable than synthetic surfactants.^3^ The structure of biosurfactants compared with conventional surfactants can differ dramatically, which gives them quite different properties, such as spontaneous curvature, critical micelle concentration (cmc), and ability to solubilize lipids.^4^ Surfactants with a high spontaneous curvature will form small micelles with relatively few monomers per micelle.^5,6^ When different amphiphilic compounds are mixed, they can self-assemble to form mixed aggregates, for example, when mixing phospholipids and surface-active compounds.^7–9^

MEDI7219 is a glucagon-like peptide (GLP-1) analogue with the purpose of treating type 2 diabetes and obesity and specifically developed to have improved bioavailability following oral administration. The peptide has 30 amino acids, six of which are acidic and expected to be deprotonated at neutral pH. On the lysine residues at positions 13 and 25, C_11_ lipids are attached via γ glutamic acid-PEG-linkers (Figure 1).^10^

**Figure 1:**
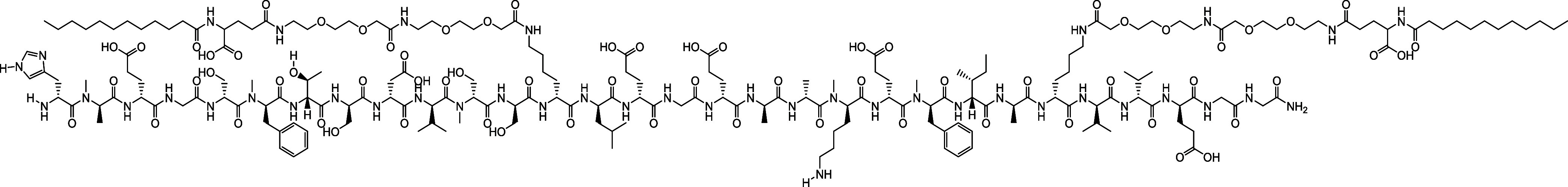
Chemical structure of MEDI7219. The peptide backbone is bis-lipidated with dodecanoic acid at the lysine residues at positions 13 and 25.

The hydrophilic and negatively charged amino acid residues of MEDI7219 together with the hydrophobic bis-lipidation give the molecule an amphiphilic structure, indicating that it could possibly self-assemble into micelles or bilayers. Self-assembly of single-tailed lipopeptides into micelles has recently been studied using small-angle X-ray scattering (SAXS).^11,12^ Lipidated peptides are gaining increased interest within the pharmaceutical industry as lipidated analogues of endogenous peptides may offer superior desirable pharmacokinetic properties compared with a nonlipidated analogue.^13,14^ Lipid conjugation of peptides can provide an albumin-binding domain, which allows longer circulation time and less frequent administration.^15^ Lipid conjugation has been observed to increase the peptide binding affinity to albumin for several GLP-1 analogues, including MEDI7219.^13,16,17^ Lipidated peptides are thus more likely to form larger complexes, as a result of either self-assembly, complexation with biological components, or albumin-binding, which will affect the transport properties of the peptide following subcutaneous administration. Larger particles, such as proteins or peptide complexes, diffuse slower; thus, the absorption from the injection site to the capillary will be slower. Particles above a certain size may also be too large to be absorbed via blood capillaries and instead absorbed via the lymphatic route. This will affect the onset of action and may have implications for bioavailability.^15,18,19^ Aggregation of peptides will not only affect the diffusion rate, but uncontrolled aggregation can also be problematic during production, induce an immunogenic response, and affect therapeutic efficacy after administration.^20^ On the other hand, controlled aggregation into e.g., oligomers or nanotubes can be utilized to obtain extended-release formulations.^21,22^ Developing methods to analyze the structure of peptide aggregates is an important step in understanding the mechanisms of aggregation and how it can be prevented or used.^23^ Self-assembly into oligomeric species of other lipidated GLP-1 analogues, such as liraglutide, has been observed previously.^24,25^ The properties of the conjugated lipid have also been investigated for analogues of liraglutide and semaglutide, suggesting a longer lipid increases the oligomerization propensity, whereas a diacid moiety reduces the oligomerization propensity.^16^

Understanding the self-assembly behavior of peptide drugs and analyzing the structures they form in solution can help understand how they interact with other compounds and materials, such as membranes, assess stability in a pharmaceutical formulation, and transport mechanisms from the injection site after administration. Much research is currently devoted to lipid-conjugated peptides, as they offer desirable properties as therapeutic agents, making it highly relevant to develop methods to study their self-assembly mechanisms. In this study, MEDI7219 is used as a model compound to investigate the aggregation mechanisms of lipidated peptide drugs in solution using small-angle X-ray (SAXS) and light scattering. The effects of varying ionic strength, pH, and buffer agents on peptide self-assembly structure are also investigated. In addition, we explore the amphiphilic character and surface-active properties of the lipidated peptide, considering it as an alternative to conventional surfactants.

## Experimental Section

All measurements were performed at 20-21 °C, and the mixed samples were stored at 21 °C and protected from light. All measurements were performed within 1 week after sample preparation. All solvents were prepared using type 1 water from a Millipore Synergy water purification system (Merck, Germany).

MEDI7219 (HαAEGSαFTSDVαSSKLEGEAAαKEαFIAKVVEGG-amide has five α-methyl amino acids and C_11_ lipids attached via γ glutamic acid-PEG-linkers at K) was provided by AstraZeneca (Gothenburg, Sweden) as a lyophilized powder. The purity of the present batch of MEDI7219 was 97.5%, as determined by RP-HPLC. Sodium thiocyanate (NaSCN), sodium chloride (NaCl), D-sorbitol, sodium acetate, glacial acetic acid, sodium phosphate dibasic heptahydrate, and sodium phosphate monobasic were all purchased from Sigma-Aldrich (Saint Louis, MO).

### Surface Tension

Surface tension was measured by using a Sigma703D Force Tensiometer (Biolin Scientific, Sweden) with the Wilhelmy plate method. All surface tension measurements were performed at 21 °C.

### Dynamic Light Scattering

Dynamic light scattering (DLS) measurements were performed on an ALV/CGS-3/MD/4 multidetection goniometer system with ALV/LSE/5004 light scattering electronics correlator (ALV, Germany), complete with a Cobolt Samba 50 mW DPSS laser 532 nm (Cobolt, Sweden) and fiber-optical single detecting units with fiber-based beam splitter for wavelength of 532 nm. Dynamic light scattering was used to determine the hydrodynamic radius of peptide aggregates and was measured at 90° for 2 min at 21 °C, 30 °C, and 37 °C. The temperature was controlled with a Julabo F25-ME heater circulator (Seelbach, Germany).

### Small-Angle X-ray Scattering

Small-angle X-ray scattering measurements were performed at the UK national synchrotron science facility Diamond Light Source at beamline B21 (Didcot, U.K.). X-rays were delivered through a bending source magnet (λ = 0.9537 Å) with a flux of 4 × 10^12^ photons per second. The scattering was detected by an EigerX 4 M X-ray detector (Dectris, Switzerland) at a sample-to-detector distance of 3688.3 mm, giving a scattering vector *q* ranging from 0.0045 to 0.34 Å^–1^. The scattering data were set to absolute intensity, and background subtracted before data analysis in the XSACT 2.4 software (Xenocs, France).

## Results and Discussion

### Critical Micelle Concentration

The critical micelle concentration (cmc) of MEDI7219 was determined by measuring the surface tension of solutions of MEDI7219 in water and 150 mM NaCl, respectively. Sodium chloride was added to mimic physiological conditions. A decrease in surface tension (γ) is observed above a certain concentration, reaching a plateau, indicating the cmc of MEDI7219 to be approximately 0.05 mg cm^–3^ (11 μM) in water and 5 × 10^–4^ mg cm^–3^ (110 nM) in 150 mM NaCl (Figure 2). The values are significantly lower than observed for some single-tailed lipopeptides.^11,12^

**Figure 2:**
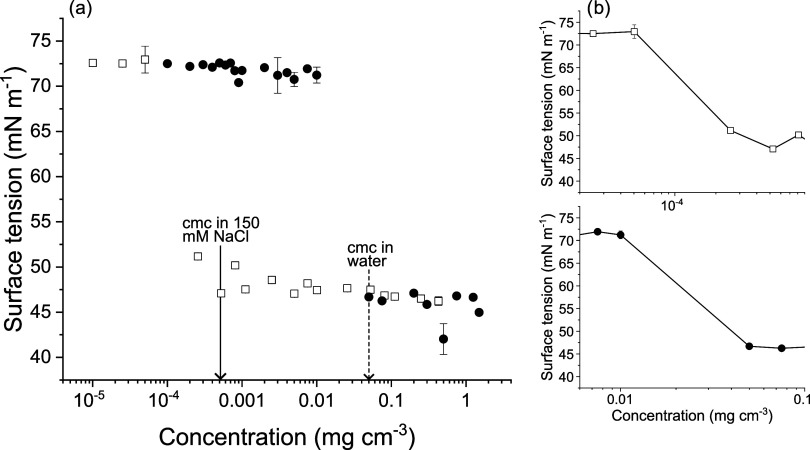
Surface tension as a function of concentration of MEDI7219 in water (●) and 150 mM NaCl solution (□). In (a), the arrows indicate where the cmc values were determined and (b) shows the surface tension around the concentration where cmc was determined. Error bars are shown for all data points and when not visible, they are within the data markers.

The surface excess (Γ) of MEDI7219 can be estimated from the slope of surface tension (γ) close to the cmc according to the Gibbs absorption isotherm

$$\Gamma =-\frac{1}{\mathrm{iRT}}{\left(\frac{\partial \gamma }{\partial \ln c}\right)}_{T}$$

where *R* is the ideal gas constant, *T* is the absolute temperature, and *c* is the concentration of surfactant. *i* is the number of ionic species per surfactant (ionic surfactant plus counterions) participating in the self-assembly process. Assuming the peptide has 6 negative charges, the value of *i* is 7. With the value of $\frac{\partial \gamma }{\partial \ln c}$ determined from the slope of the surface tension curve in Figure 2b, the surface excess and the corresponding surface area per molecule were estimated to 230 Å^2^ and 260 Å^2^ per molecule in water and 150 mM NaCl, respectively. These values are slightly larger than what has previously been observed for the (C_18_-lipid) lipopeptide surfactin, which is a smaller molecule.^26^ However, if the p*K*_a_ values, thus the surface charge, change when the peptides pack at the surface, the value of *i* and the estimated area per surfactant would decrease.

The cmc of an ionic surfactant is expected to decrease when salt is added to the solvent, since the entropic loss of recruiting counterions to screen the repulsion between the charged head groups decreases with increasing electrolyte concentration. Our obtained cmc values are of the same order of magnitude as previously recorded for the lipidated GLP-1 analogue liraglutide, and surfactin.^25,27–29^ The slightly lower cmc values obtained for MEDI7219, compared with liraglutide and surfactin, are likely related to the larger hydrophobic group of MEDI7219. MEDI7219 has two C_11_-lipids, liraglutide has one C_15_-lipid, and surfactin has one C_11_-lipid.

The surface tension decreases from 72 mN m^–1^ to about 45 mN m^–1^, which is a smaller decrease than observed for the conventional ionic surfactant sodium dodecyl sulfate (SDS) and the lipopeptide surfactant surfactin.^30,31^ The surface-active properties of surfactants with a highly charged bulky headgroup, like MEDI7219, are expected to be lower compared to smaller surfactants.^32^

The lowest concentration of MEDI7219 used when analyzing the structure of self-assembled peptide aggregates was 1.0 mg cm^–3^, which is 20-fold higher than the cmc of MEDI7219 in water. This means that, in our samples studied with SAXS, the majority of peptides are located in micelles and that the free monomer concentration is expected to be relatively low.

### Dynamic Light Scattering and Small-Angle X-ray Scattering

MEDI7219 was dissolved in six aqueous solvents with a peptide concentration of 1–50 mg cm^–3^ (corresponding to 0.23–11.5 mM). The solvents were water, 150 mM NaCl solution, 150 mM NaSCN solution, 20 mM phosphate buffer pH 7.5 with and without 200 mM sorbitol, and 68 mM acetate buffer, pH 5.7. The buffer concentrations of the phosphate and acetate buffers were selected to result in the same ionic strength at the chosen pH values. The buffer types and the chosen pH values were based on what is commonly used in pharmaceutical formulations. NaSCN was chosen to compare the differences caused by adding salts from different ends of the Hofmeister series.^33,34^ Sorbitol was added as it is commonly used as a tonicity agent in liquid pharmaceutical formulations.^35^

The peptide dissolved and formed perfectly clear, transparent solutions in all solvents and at all peptide concentrations that were used. Dynamic light scattering suggests that the particles formed by MEDI7219 in 150 mM NaCl are about 2–3 nm in hydrodynamic radius. In water, the micelles appear somewhat smaller as a result of the stronger repulsive intermicellar forces causing faster apparent diffusion of the micelles (Figure 3).

**Figure 3:**
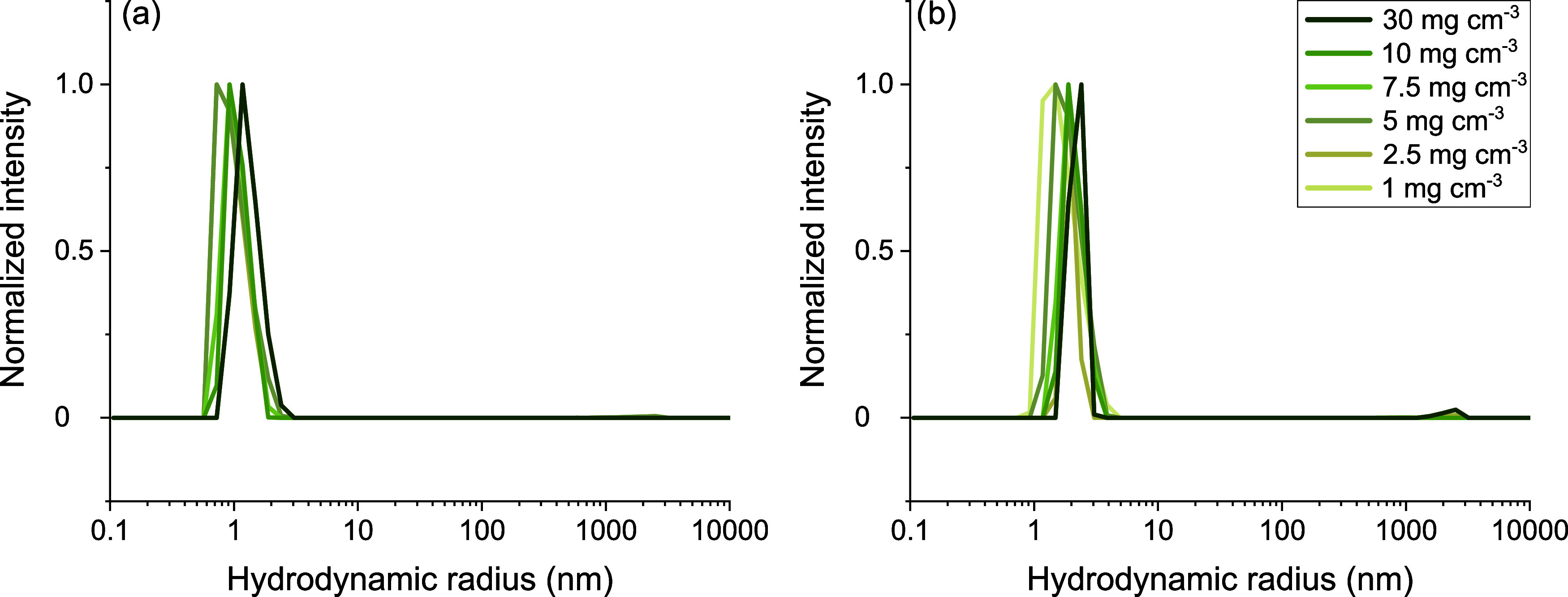
Mass weighted distribution of hydrodynamic radius obtained from DLS for (a) 2.5–30 mg cm^–3^ MEDI7219 in water and (b) 1–30 mg cm^–3^ in 150 mM NaCl.

The effect of temperature on the size of self-assembled MEDI7219 aggregates was also investigated by DLS. The distribution of *R*_h_ of MEDI7219 in water and 150 mM NaCl did not change when the temperature was increased from 21 to 37 °C (Figure S1).

Small-angle X-ray scattering data for all samples are shown in Figure 4. The intensity as a function of scattering vector *q* displays the characteristic oscillation at high *q*-values of core-and-shell particles. The presence of a Guinier regime at comparatively high *q*-values indicates a small particle size. The scattering profiles of MEDI7219 in all different aqueous solutions appear to be rather similar. For samples with MEDI7219 in pure water and phosphate buffer (with and without sorbitol), a slight decrease in scattering intensity at low *q*-values could be observed, indicating significant structure factor effects caused by repulsive interparticle interactions. In pure water and 20 mM phosphate buffer, the ionic strength is not sufficiently high to significantly screen the interparticle repulsion between the charged particles. When 150 mM NaCl and NaSCN, respectively, are added to the solvent, there is no effect of interparticle interactions appearing in the scattering data.

**Figure 4:**
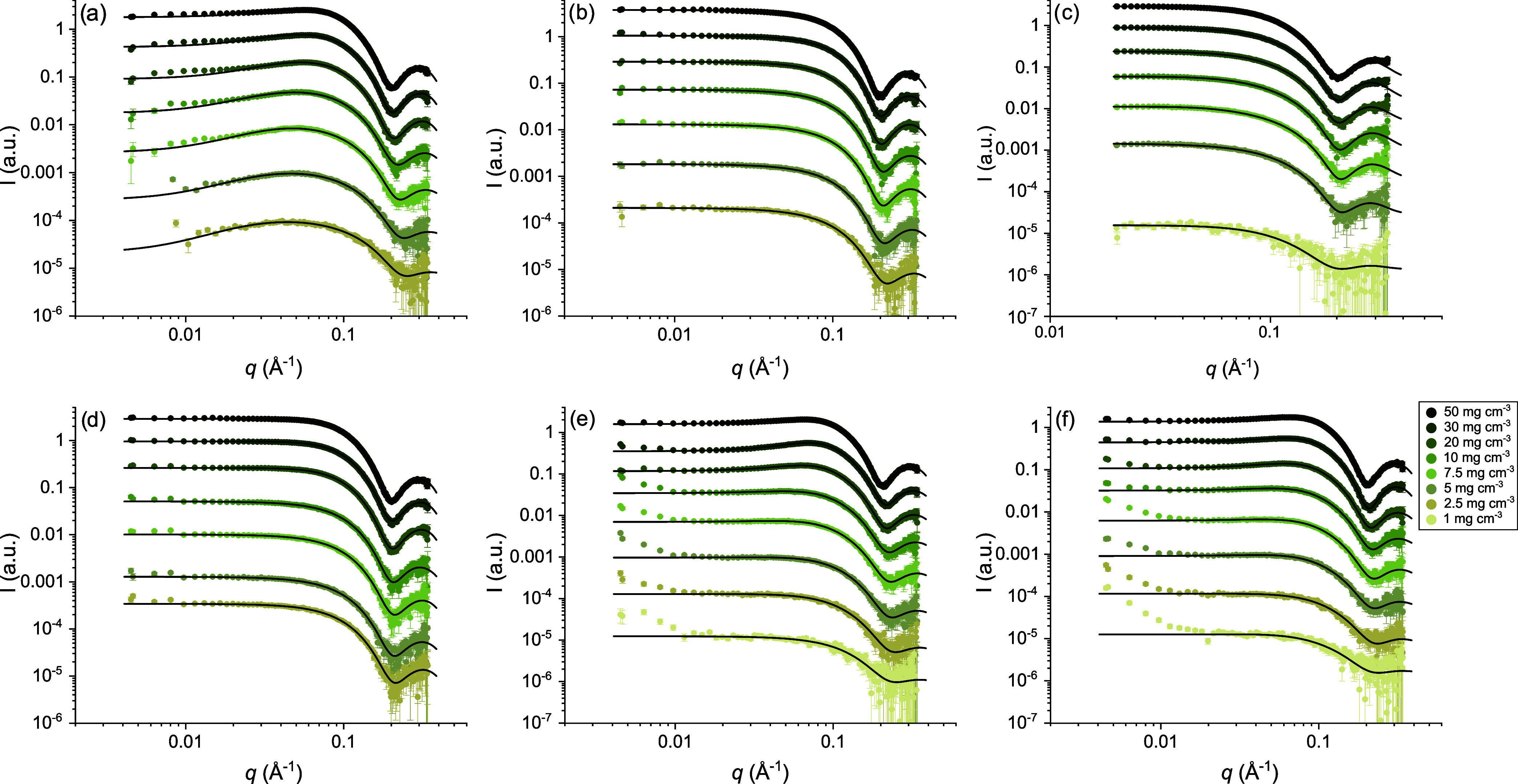
Scattering intensity as a function of scattering vector *q* for 1–50 mg cm^–3^ MEDI7219 in (a) water, (b) 150 mM NaCl, (c) 150 mM NaSCN, (d) acetate buffer, pH 5.7, (e) phosphate buffer, pH 7.5, and (f) phosphate buffer, pH 7.5, with 200 mM sorbitol fitted with a core-and-shell triaxial ellipsoid model (solid line). The quality of data after buffer subtraction for the lowest peptide concentrations in some buffers was not sufficiently high for analysis and is not included in Figure 4. The *q*-range for samples in 150 mM NaSCN (c) differs from the rest, due to insufficient data quality at low *q* after background subtraction. The data in all subfigures have been offset for clarity by multiplying the absolute intensity by 0.0033, 0.0125, 0.05, 0.25, 1, 2, 5, and 10 for concentrations 1–50 mg cm^–3^, respectively.

### Analysis of Small-Angle X-ray Scattering Measurements

Small-angle X-ray scattering data were analyzed by means of least-squares model fitting. The best agreement with all data was found using a model for core-and-shell triaxial general ellipsoids (Figure 5).

**Figure 5:**
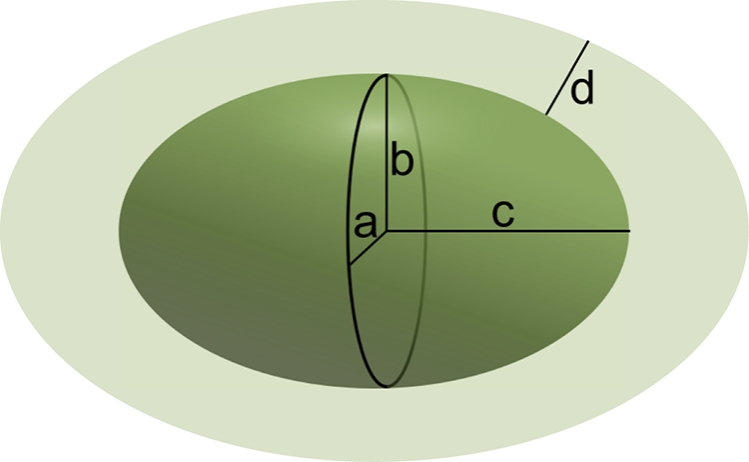
Illustration of a triaxial core-and-shell ellipsoid with the core dimensions expressed as half axes *a*, *b*, and *c* and shell thickness *d*.

This model gives significantly better agreement with data as compared to models of the biaxial ellipsoid of revolution and sphere, respectively. The model is described in detail in the Supporting Information, and the results of the data analysis are given in Figure 6 and Table S1. Triaxial tablet-shaped micelles have previously been observed with small-angle scattering in a number of surfactant systems, and they have been theoretically rationalized in the general micelle model.^6,9,36,37^

**Figure 6:**
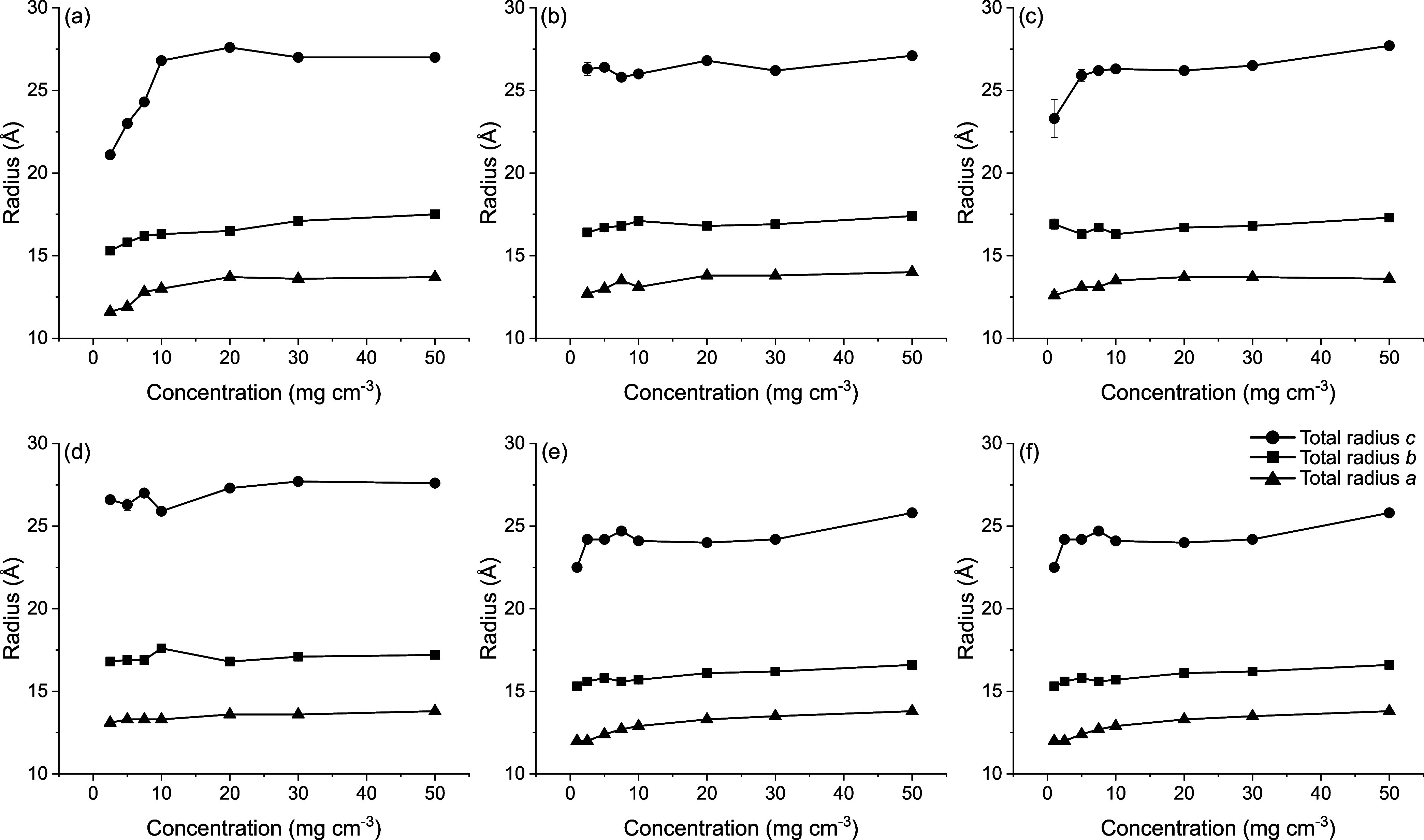
Total radii (half-axis and shell thickness) of MEDI7219 micelles obtained from model fitting with a core-and-shell triaxial ellipsoid. The radius along the three axes of the ellipsoids *a* (▲), *b* (■), and *c* (●) for 1–50 mg cm^–3^ MEDI7219 in (a) water, (b) 150 mM NaCl, (c) 150 mM NaSCN, (d) acetate buffer, pH 5.7, (e) phosphate buffer, pH 7.5, and (f) phosphate buffer, pH 7.5, with 200 mM sorbitol. Error bars are shown for all data points and when not visible they are within the data markers.

Our SAXS data analysis demonstrates that MEDI7219 in the various aqueous solutions forms small general ellipsoidal micelles with a maximum half-axis of the length dimension equal to about 20 Å and 7.5 Å shell thickness.

The increase in scattering intensity at *q* < 0.01 Å^–1^ observed for samples in phosphate buffer at low concentrations indicates the presence of larger particles, possibly indicating aggregation into larger structures below 7.5 mg cm^–3^. Nevertheless, the scattering from micelles at higher *q* is predominant at all concentrations in phosphate buffer, demonstrating that larger particles are present in only very low amounts.

Assuming that the ellipsoidal core consists of only the hydrophobic lipid tails attached to the peptide allows us to calculate the number of MEDI7219 molecules in the micelles. The volume of the two lipids was calculated as the sum of two C_11_ saturated aliphatic hydrocarbon chains (647 Å^3^).^38^ The aggregation number (*N*_agg_) varies slightly with peptide concentration and is around 5–8 in the solvents where salt is present (Figure 7).

**Figure 7:**
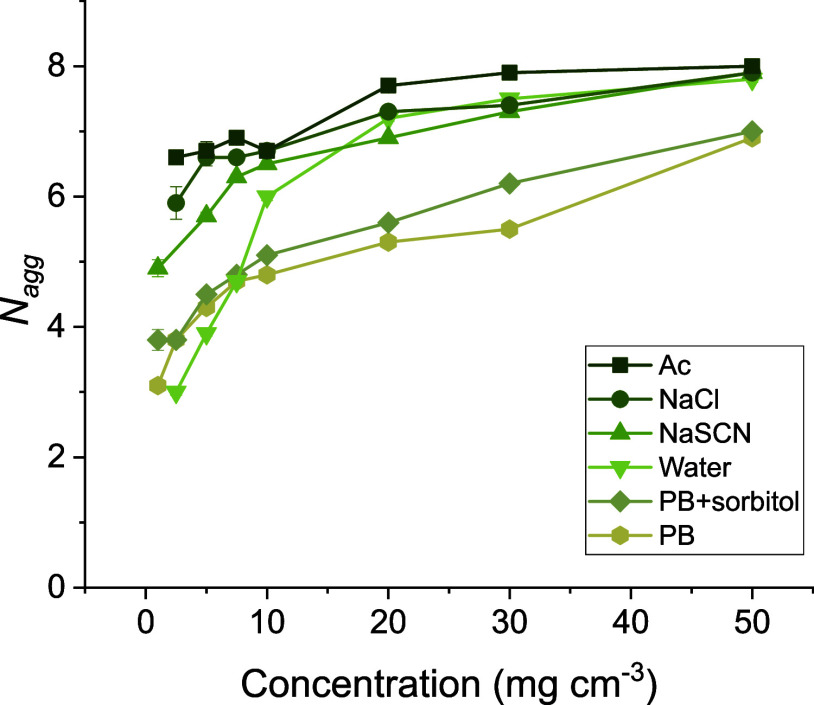
Aggregation number (*N*_agg_) of 1–50 mg cm^–3^ MEDI7219 micelles in acetate buffer (■), 150 mM NaCl (●), 150 mM NaSCN (▲), water (▼), phosphate buffer (PB) with 200 mM sorbitol (⧫), and phosphate buffer (PB) (⬢). Error bars are shown for all data points and when not visible they are within the data markers.

In pure water, the micelles appear comparatively small at the lowest peptide concentration, followed by a characteristic growth behavior with increasing concentration.^6^ The micelles are also significantly smaller in phosphate buffer than in the other solvents. At the higher pH (7.5) in the phosphate buffer, the acidic amino acid residues of MEDI7219 are expected to be fully deprotonated and negatively charged, implying a larger surface charge density of the micelles. The electrostatic repulsion between the MEDI7219 head groups increases with higher charge, which increases the spontaneous curvature of MEDI7219, making the micelles smaller. The slightly larger micelle size observed for MEDI7219 micelles in acetate buffer (pH 5.7) can be explained by a similar mechanism, giving a reduced surface charge density of the micelles. We may, however, note that the p*K*_a_ values of the acidic amino acid residues in MEDI7219 are likely affected by the process of packing peptides in micelles.^39^

The aggregation numbers of MEDI7219 are generally much smaller than those for most conventional surfactants, indicating a very high spontaneous curvature of the peptide surfactant, despite having two aliphatic hydrocarbon chains as tails. The spontaneous curvature of surfactants is expected to decrease with increasing lipid tail length as well as increasing number of tails.^40^ MEDI7219 has shorter fatty acid chains than other GLP-1 analogues (such as liraglutide, semaglutide, or tirzepatide), which have one C_16_/C_18_ chain, but the total number of carbons in the lipid tails is larger for MEDI7219 than in any of the other analogues.

The size and shape of the peptide surfactant micelles appear to be surprisingly insensitive to the choice of solvent. The size of a micelle consisting of ionic surfactants may occasionally be influenced by the electrolyte concentration. The ions from the added electrolyte will screen the repulsion between the charged head groups, and as a result, the effective area per headgroup as well as the curvature of the micelle interface decreases and the aggregation number increases. For some conventional surfactants, like sodium dodecyl sulfate (SDS) and cetyltrimethylammonium bromide (CTAB), this effect is shown as a greatly increased aggregation number when salt is added, which demonstrates the presence of a second cmc where small globular or ellipsoidal micelles start to grow strongly in the length direction above a certain concentration.^41–43^ However, in the case of considerably charged MEDI7219, this effect was not observed.

Hence, we may conclude that MEDI7219 lacks a second cmc within the measured range of concentrations in the different solvents. The conspicuously weak growth behavior of the micelles demonstrates that they must be close to monodisperse, in accordance with the relation derived by Hall and Pethica.^6,44^ This is consistent with our SAXS data analysis, according to which the data were best fitted with a model for monodisperse ellipsoids.

For conventional surfactants, the first and second cmc usually follow one another. For instance, many nonionic surfactants frequently display low first as well as second cmc. In contrast, the peptide drug surfactant MEDI7219 has a very low first cmc, and if a second cmc exists, it is much higher than the first cmc, resembling the behavior of bile salt surfactants.^9^ The insensitivity with respect to pH and electrolyte concentration indicates that the size of the bulky peptide headgroup of MEDI7219 contributes significantly to the large spontaneous curvature of the peptide surfactant.

Notably, the small aggregation numbers of MEDI7219 are similar in magnitude to those observed for bile salt surfactant micelles. Bile salt molecules have a rigid structure, and the hydrophobic and hydrophilic moieties are separated to different sides of the molecule, forming a comparatively flat two-face amphiphilic molecule, in contrast to the head–tail structure of conventional surfactants. Bile salt surfactant micelles are typically made up of much fewer monomers than conventional surfactants and have significantly higher interfacial curvature.^45^ The high spontaneous curvature of the surfactants makes them efficient at dissolving lipid bilayers.^9^ MEDI7219 does have the general head–tail structure but forms small micelles with relatively few monomers, indicating a large spontaneous curvature, in similarity with bile salt surfactants. This suggests that MEDI7219 could be efficient at dissolving lipids, and bis-lipidated peptides are efficient as novel surfactants with the possibility of affecting lipid membrane structure.

The shell thickness of the micelles is similar at all peptide concentrations and does not vary in different buffers. The shell of the micelles is expected to consist of the peptide headgroup together with water. The shell-to-core ratio of scattering length density contrast, Δ*ρ*_shell/_ Δ*ρ*_core_, was determined from our SAXS data analysis (Table S1) and is found to be very close to values estimated from calculated scattering length densities for headgroup and tails, respectively. Hence, we may conclude that the peptide head groups are rather closely packed in the micelle shell with low hydration and low contribution from the counterions. The volume of a MEDI7219 headgroup (including the linker moiety) was estimated based on the volume contribution of each chemical group in water to 5069 Å^3^, whereas the total volume of the two lipids is 647 Å^3^.^46^ The volume of the shell as obtained from our data analysis is found to be about five times larger than the volume of the core. This is somewhat lower than the estimated peptide-to-lipid volume ratio, which is 6–8 depending on whether the hydrophilic linker is included or not in the headgroup. Hence, the comparatively thin shell thickness indicates that substantial parts of the peptide/linker head groups are not included in the shell, probably due to low contrast with the aqueous bulk phase. Most likely, the hydrophobic moieties of the peptide groups are attached to the lipid core, whereas hydrophilic/charged groups, with a hydration layer, are located at the micelle-to-water interface. Since most of the water adsorbed to the peptide is located at the micelle interface, in a layer with low contrast, the shell thickness as obtained from our data analysis appears rather thin.

The linker moiety between the headgroup and the lipids includes some uncertainty for the distinction between core and shell. It is, however, difficult to precisely calculate the molecular volume of the considerably charged peptide group and estimate how it might change upon micellization. The aggregation numbers shown in Figure 7 were calculated based on the core dimensions and well-known molecular volumes of lipid hydrocarbons.^47^ The shielding of the hydrocarbon–water interface by the bulky peptide head-groups of MEDI7219 may explain the very small sizes of the micelles.^48^

The seemingly very low first cmc of MEDI7219, comparable with peak plasma concentrations of other GLP-1 analogues, suggests that the micelle structure may be retained even at therapeutic concentrations in the blood.^49^ If micelles are retained in the blood, it may offer an alternative or complementary explanation to the already proposed albumin-lipidated peptide complexes suggested to be the main reason for the extended circulation time of therapeutic lipidated peptides. However, the self-assembly and micellization of MEDI7219 could be affected by plasma components, such as albumin, which warrants further investigation in the presence of additional biological components. Nevertheless, the aggregation number of MEDI7219 micelles is in good agreement with hexa-octameric species that have been observed for other GLP-1 analogues.^24,25^

## Conclusions

We have demonstrated that the lipidated peptide drug MEDI7219 behaves as a surfactant and self-assembles above the critical micelle concentration to small ellipsoidal micelles with aggregation numbers less than 10, in various aqueous solvents. In terms of aggregation number, the peptide surfactant micelles appeared much smaller than micelles formed by conventional surfactants, indicating a very high spontaneous curvature, despite being composed of two aliphatic chains as a hydrophobic part. The critical micelle concentration of MEDI7219 was found to be very low, and the size of the formed micelles appeared surprisingly insensitive to environmental conditions such as type of electrolyte, pH, type of buffer, and temperature. The low critical micelle concentration of MEDI7219 suggests it could solubilize hydrophobic compounds in its core or oppositely charged compounds near the shell, even at low concentrations. The distinct surfactant properties and high spontaneous curvature indicate that MEDI7219 could interact with other amphiphilic compounds, e.g., phospholipids, to form mixed micelles, similar to other surfactants including bile salts.^7–9^

The results of this study suggest that lipidated peptides can be an alternative to conventional surfactants in the search for efficient and biodegradable surfactants. Moreover, if a peptide forms aggregates after subcutaneous administration, it may impact the absorption from the extracellular space due to size limitations for absorption via capillaries.^18^ This will impact the pharmacokinetic profile of the drug, and must be taken into account when studying the therapeutic effects after administration.^19^

## Supplementary Material



## Notes

Information about the model used to analyze small-angle X-ray scattering data, additional DLS results, and a table with the fitted parameters. (PDF)
