# Supplementary material for: Self-Assembly of a Therapeutic Peptide Surfactant: A Small-Angle X‑ray Scattering Study
Source: Langmuir. 2026 Feb 20;42(8):6471–8. doi: 10.1021/acs.langmuir.5c06529 (PMC12961922; doi:10.1021/acs.langmuir.5c06529)
Supplement: Supplementary file 1 [file la5c06529_si_001.pdf]

Supplementary material: Self-assembly of a therapeutic peptide surfactant: A small-angle X-ray scattering study

Ellen Brunzell[a*], Kalle Sigfridsson[b], and L. Magnus Bergström[a]
[a]Department of Medicinal Chemistry, Pharmaceutical Physical Chemistry, Uppsala University, 751 23 Uppsala, Sweden.
[b]Advanced Drug Delivery, Pharmaceutical Science, R&D, AstraZeneca, 431 83 Gothenburg, Sweden.

[*]ellen.brunzell@ilk.uu.se

Contents

- I. Model employed in the least-square fitting analysis of small-angle X-ray scattering data
- II. Additional DLS measurements and results from model fitting of SAXS data

I. Model employed in the least-square fitting analysis of small-angle X-ray scattering data

The best fit of the micelles was achieved using a form factor P(q) for core-shell triaxial ellipsoid with axes a, b, and c and shell thickness d. The Hayter-Penfold rescaled mean spherical approximation (MSA) was used as a structure factor S(q)[1,2] in a decoupling approximation.[3] For monodisperse micelles, the scattering cross section as a function of scattering vector, q, can be described as[4]

$$\frac{d\sigma(q)}{d\Omega} = n(\Delta\rho_{core}V_{core})^2 P(q)\left[1+\beta(q)(S(q)-1)\right] \quad \text{Eq. S1}$$

where n is the number density of particles and

$$P(q) = \langle F^2(q)\rangle = \frac{2}{\pi}\int_0^{\pi/2}\int_0^{\pi/2} F(q,\phi,\theta)^2 \sin\phi \, d\phi d\theta \quad \text{Eq. S2}$$

where $F(q)$ is the amplitude of the form factor and

$$\beta(q) = \frac{\langle F(q)\rangle^2}{\langle F^2(q)\rangle} \quad \text{Eq. S3}$$

where

$$\langle F(q)\rangle = \frac{2}{\pi}\int_0^{\pi/2}\int_0^{\pi/2} F(q,\phi,\theta)\sin\phi \, d\phi d\theta \quad \text{Eq. S4}$$

For a core-and-shell micelle, the amplitude can be written as a sum of contributions from the core and shell, respectively,

$$F(q,\phi,\theta) = F_{core}(q,\phi,\theta) + \varrho\frac{V_{shell}}{V_{core}}F_{shell}(q,\phi,\theta) \quad \text{Eq. S5}$$

Where

$$\varrho = \frac{\Delta\rho_{shell}}{\Delta\rho_{core}} \qquad \text{Eq. S6}$$

$\Delta\rho_{core}$ and $\Delta\rho_{shell}$ are the differences in scattering length densities between core and solvent, and shell and solvent, respectively. The volume of the core is $V_{core} = V(a, b, c)$ and the volume of the shell is $V_{shell} = V(a + d, b + d, c+d) - V(a, b, c)$.
For a general ellipsoid with axes a, b, and c the amplitude as a function of the radial distance, r, is

$$F_{core}\big(qr(a,b,c,\phi,\theta)\big) = F_{sph} \qquad \text{Eq. S7}$$

where

$$F_{sph} = \frac{3[\sin(qr) - qr\cos(qr)]}{(qr)^3} \qquad \text{Eq. S8}$$

with

$$r(a,b,c,\phi,\theta) = \sqrt{(a^2\sin^2\theta + b^2\cos^2\theta)\sin^2\phi + c^2\cos^2\phi} \qquad \text{Eq. S9}$$

The corresponding quantity for the shell is described as

$$F_{shell} = \frac{V_{tot}F_{sph}\big(q,r(a+d,b+d,c+d,\phi,\theta)\big) - V_{core}F_{sph}\big(q,r(a,b,c,\phi,\theta)\big)}{V_{shell}} \qquad \text{Eq. S10}$$

where $V_{tot} = V(a + d, b + d, c + d) = V_{core} + V_{shell}$ and

$$r(a+d,b+d,c+d,\phi,\theta) = \sqrt{((a+d)^2\sin^2\theta + (b+d)^2\cos^2\theta)\sin^2\phi + (c+d)^2\cos^2\phi}$$
Eq. S11

The fitted parameters in the model were optimized by conventional least-square analysis, and the errors were calculated with conventional methods.[4,5] The reduced χ^2 parameter was used to determine the quality of the fit

$$\chi^2 = \frac{1}{N-M}\sum_{i=1}^{N}\left(\frac{I_{exp}(q_i) - I_{mod}(q_i)}{\sigma_i}\right)^2 \qquad \text{Eq. S12}$$

where $I_{exp}(q_i)$ and $I_{mod}(q_i)$ are the experimental and model intensities, respectively, at scattering vector modulus, q_i, σ_i represent the statistical uncertainties of the data points, N is the total number of data points, and M is the number of optimized fitted parameters.

Additional measurements and results from model fitting of SAXS data

The hydrodynamic radius of MEDI7219 in water and 150 mM NaCl was determined at 21 °C, 30 °C, and 37 °C to determine the effect of temperature on micelle size.

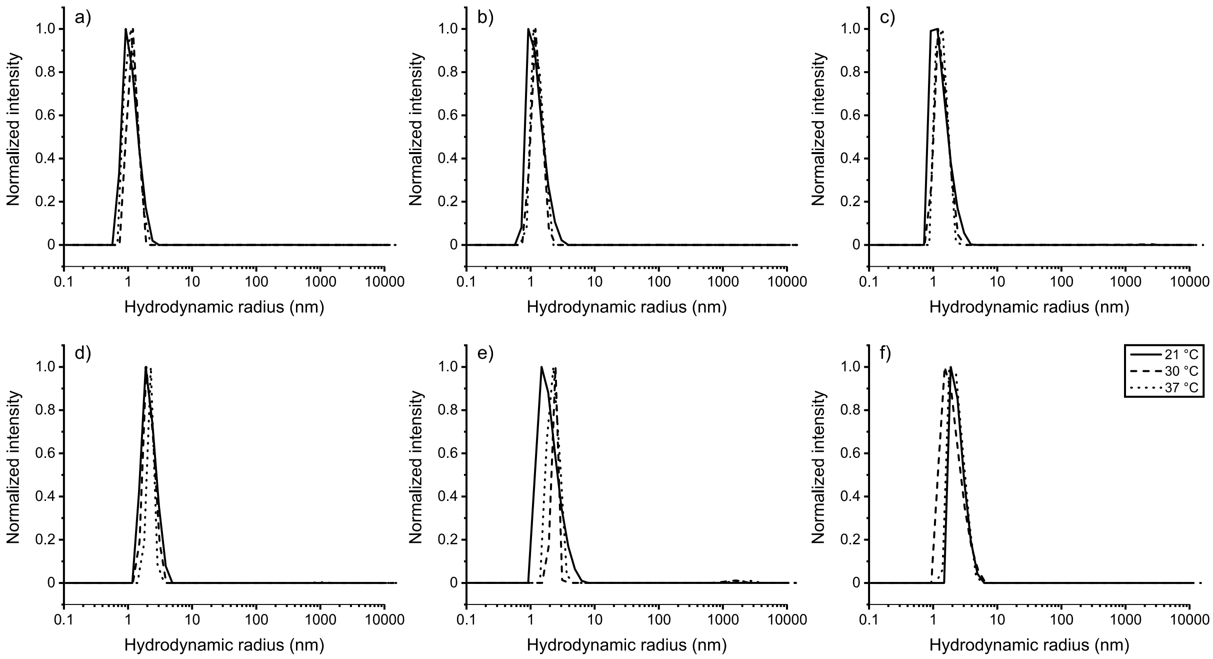

Figure S1. Mass weighted distribution of hydrodynamic radii for micelles formed by a) 10 mg cm^{-3}, b) 20 mg cm^{-3} and c) 30 mg cm^{-3} MEDI7219 in water and d) 10 mg cm^{-3}, e) 20 mg cm^{-3} and f) 30 mg cm^{-3} MEDI7219 in 150 mM NaCl at 21 °C (solid line), 30 °C (- - -), and 37 °C (···) determined by DLS.

The scattering length density (SLD) of the MEDI7219 head group was estimated to $\mathrm{SLD}_{7219,\,\mathrm{head}} = 12 \times 10^{-6}\text{Å}^{-2}$ and the volume used was 5069 Å^3, the SLD for the MEDI7219 C_{11} lipids was estimated to $\mathrm{SLD}_{7219,\,\mathrm{lipids}} = 8.54 \times 10^{-6}\text{Å}^{-2}$ and the volume used was 647 Å^3, and the SLD of water is $\mathrm{SLD}_{water} = 9.43 \times 10^{-6}\text{Å}^{-2}$. The fitted parameter $\Delta\rho_{shell}/\Delta\rho_{core}$ suggests that the shell consists of approximately 95% peptide (head group) and 5% water.

If the aggregation number is calculated from the total micelle volume and assuming the full peptide volume is 5716 Å^3, the number of monomers per micelle is approximately 3-5, which is lower than the estimated values based on the core dimensions. The aggregation number calculated from the absolute forward scattering intensity is approximately 3-4 monomers per micelle. A weakly scattering hydration layer at the micelle interface that may incorporate hydrophilic/charged side groups of the peptide will contribute weakly to the scattering intensity, which may explain why the aggregation number estimates slightly differ when calculated from core dimensions, total micelle volume and absolute scattering intensity, respectively.

Table S1. Fitting parameters of SAXS measurements on 1-50 mg/ml MEDI7219 in water, 150 mM NaCl and NaSCN, acetate buffer, phosphate buffer, and phosphate buffer with 200 mM sorbitol.

**Buffer**	**Conc. (mg/ml)**	**Half-axis *a* (Å)**	**Half-axis *b* (Å)**	**Half-axis c (Å)**	**Shell thickness (Å)**	N_{agg}	$\Delta\rho_{shell}/\Delta\rho_{core}$ [1]	**Effective charge**	**Volume fraction**[2]
Water	2.5	4.2±0.02	7.9±0.04	13.7±0.1	7.4±0.01	3.0±0.05	-1.46±0.01	4.7±0.2	0.00026
Water	5	4.6±0.01	8.5±0.02	15.7±0.08	7.3±0.01	3.9±0.04	-1.48±0.01	4.5±0.1	0.00070
Water	7.5	5.1±0.01	8.5±0.02	16.6±0.11	7.7±0.01	4.7±0.05	-1.49±0.01	4.6±0.1	0.00108
Water	10	5.5±0.01	8.8±0.02	19.3±0.12	7.5±0.01	6.0±0.06	-1.49±0.01	5.0±0.1	0.00143
Water	20	6.2±0.01	9.0±0.01	20.1±0.08	7.5±0.01	7.2±0.05	-1.49±0.01	5.1±0.1	0.00287
Water	30	6.1±0.01	9.6±0.02	19.5±0.09	7.5±0.01	7.5±0.07	-1.49±0.01	4.5±0.1	0.00430
Water	50	6.2±0.01	10.0±0.02	19.5±0.07	7.5±0.01	7.8±0.05	-1.49±0.01	4.0±0.1	0.00714
150 mM NaCl	2.5	5.3±0.05	9.0±0.12	18.9±0.33	7.4±0.06	5.9±0.25	-1.46±0.01	0.2±108	0.00035
150 mM NaCl	5	5.7±0.03	9.4±0.07	19.1±0.15	7.3±0.02	6.6±0.13	-1.48±0.01	17.3±1.8	0.00071
150 mM NaCl	7.5	6.0±0.02	9.3±0.01	18.3±0.09	7.5±0.01	6.6±0.09	-1.48±0.01	14.7±0.8	0.00105
150 mM NaCl	10	5.7±0.01	9.7±0.01	18.6±0.03	7.4±0.01	6.7±0.02	-1.48±0.01	16.3±0.3	0.00141
150 mM NaCl	20	6.3±0.01	9.3±0.03	19.3±0.03	7.5±0.01	7.3±0.05	-1.46±0.01	14.2±0.2	0.00281
150 mM NaCl	30	6.4±0.02	9.5±0.05	18.8±0.03	7.4±0.01	7.4±0.07	-1.48±0.01	11.6±0.2	0.00424
150 mM NaCl	50	6.4±0.01	9.8±0.01	19.5±0.02	7.5±0.01	7.9±0.02	-1.49±0.01	7.9±0.1	0.00566
150 mM NaSCN	1	5.1±0.09	9.4±0.2	15.8±1.01	7.5±0.13	4.9±0.51	-1.50±0.04	68±58	0.00045
150 mM NaSCN	5	5.5±0.01	8.7±0.02	18.3±0.33	7.6±0.04	5.7±0.13	-1.49±0.01	31.2±4	0.00074
150 mM NaSCN	7.5	5.6±0.01	9.2±0.02	18.7±0.12	7.5±0.01	6.3±0.06	-1.49±0.01	31.1±2	0.00098
150 mM NaSCN	10	6.0±0.01	8.8±0.01	18.8±0.08	7.5±0.01	6.5±0.04	-1.48±0.01	24.2±1	0.00143
150 mM NaSCN	20	6.2±0.01	9.2±0.01	18.7±0.03	7.5±0.01	6.9±0.02	-1.49±0.01	16.9±0.2	0.00283
150 mM NaSCN	30	6.3±0.01	9.4±0.01	19.1±0.02	7.4±0.01	7.3±0.02	-1.48±0.01	13.8±0.1	0.00427
150 mM NaSCN	50	6.1±0.01	9.8±0.01	20.2±0.02	7.5±0.01	7.9±0.02	-1.49±0.01	12.5±0.1	0.00699
Acetate buffer pH 5.7	2.5	5.7±0.01	9.4±0.02	19.2±0.15	7.4±0.02	6.6±0.07	-1.49±0.01	15.3±1.8	0.00032
Acetate buffer pH 5.7	5	5.8±0.01	9.4±0.02	18.8±0.32	7.5±0.03	6.7±0.14	-1.47±0.01	7.0±1.2	0.00071
Acetate buffer pH 5.7	7.5	5.8±0.01	9.4±0.01	19.5±0.11	7.5±0.01	6.9±0.06	-1.49±0.01	7.5±0.4	0.00104
Acetate buffer pH 5.7	10	5.7±0.01	10.0±0.01	18.3±0.07	7.6±0.01	6.7±0.04	-1.49±0.01	6.2±0.2	0.00146
Acetate buffer pH 5.7	20	6.3±0.01	9.5±0.01	20.0±0.02	7.3±0.01	7.7±0.02	-1.49±0.01	8.0±0.1	0.00292
Acetate buffer pH 5.7	30	6.2±0.01	9.7±0.01	20.3±0.02	7.4±0.01	7.9±0.01	-1.49±0.01	7.3±0.1	0.00455
Acetate buffer pH 5.7	50	6.3±0.01	9.7±0.01	20.1±0.04	7.5±0.01	8.0±0.03	-1.49±0.01	6.5±0.1	0.00569
PB pH 7.5	1	4.3±0.05	7.6±0.1	14.8±0.01	7.7±0.03	3.1±0.08	-1.48±0.01	7.8±6.5	0.00017
PB pH 7.5	2.5	4.4±0.02	8.0±0.04	16.6±0.14	7.6±0.02	3.8±0.06	-1.49±0.01	15.0±2	0.00034
PB pH 7.5	5	4.8±0.01	8.2±0.02	16.6±0.08	7.6±0.01	4.3±0.04	-1.49±0.01	14.3±0.5	0.00071
PB pH 7.5	7.5	5.2±0.01	8.1±0.01	17.2±0.08	7.5±0.01	4.7±0.03	-1.49±0.01	14.9±0.2	0.00107
PB pH 7.5	10	5.4±0.01	8.2±0.01	16.6±0.07	7.5±0.01	4.8±0.03	-1.49±0.01	14.8±0.1	0.00143
PB pH 7.5	20	5.8±0.01	8.6±0.01	16.5±0.04	7.5±0.01	5.3±0.02	-1.49±0.01	15.5±0.1	0.00283
PB pH 7.5	30	5.9±0.01	8.6±0.01	16.6±0.02	7.6±0.01	5.5±0.01	-1.49±0.01	14.8±0.1	0.00428
PB pH 7.5	50	6.3±0.01	9.1±0.01	18.3±0.02	7.5±0.01	6.9±0.01	-1.49±0.01	10.1±0.1	0.00715
PB pH 7.5+200 mM sorbitol	1	4.6±0.05	8.5±0.1	15.3±0.44	7.5±0.05	3.8±0.16	-1.48±0.02	31.4±21	0.00019
PB pH 7.5+200 mM sorbitol	2.5	4.8±0.02	8.4±0.05	14.7±0.16	7.6±0.02	3.8±0.08	-1.47±0.01	17.1±3	0.00033
PB pH 7.5+200 mM sorbitol	5	5.3±0.01	8.5±0.02	15.5±0.15	7.7±0.02	4.5±0.07	-1.50±0.01	18.2±1	0.00074
PB pH 7.5+200 mM sorbitol	7.5	5.4±0.01	8.4±0.02	16.1±0.09	7.7±0.01	4.8±0.05	-1.48±0.01	15.5±0.3	0.00111
PB pH 7.5+200 mM sorbitol	10	5.4±0.01	9.2±0.01	15.8±0.08	7.5±0.01	5.1±0.04	-1.49±0.01	15.9±0.1	0.00145
PB pH 7.5+200 mM sorbitol	20	6.1±0.01	8.6±0.01	16.4±0.05	7.6±0.01	5.6±0.03	-1.49±0.01	14.7±0.1	0.00286
PB pH 7.5+200 mM sorbitol	30	6.6±0.01	8.7±0.01	16.6±0.04	7.6±0.01	6.2±0.03	-1.49±0.01	11.8±0.1	0.00429
PB pH 7.5+200 mM sorbitol	50	7.6±0.01	9.0±0.01	18.0±0.02	7.5±0.01	7.0±0.02	-1.49±0.01	10.1±0.1	0.00709

[1]$\Delta\rho_{shell}$ is the difference in scattering length density between the shell (peptide head group including linker) and water and $\Delta\rho_{core}$ is the difference in scattering length density between the core (the two C_{11} lipids attached to the peptide) and water.
[2]The volume fraction was calculated from peptide concentration and kept fixed in the model fitting.

(1) Hansen, J.-P.; Hayter, J. B. A Rescaled MSA Structure Factor for Dilute Charged Colloidal Dispersions. *Molecular Physics* **1982**, *46* (3), 651–656. https://doi.org/10.1080/00268978200101471.
(2) Hayter, J. B.; Penfold, J. An Analytic Structure Factor for Macroion Solutions. *Molecular Physics* **1981**, *42* (1), 109–118. https://doi.org/10.1080/00268978100100091.
(3) Kotlarchyk, M.; Chen, S. Analysis of Small Angle Neutron Scattering Spectra from Polydisperse Interacting Colloids. *The Journal of Chemical Physics* **1983**, *79* (5), 2461–2469. https://doi.org/10.1063/1.446055.
(4) Pedersen, J. S. Analysis of Small-Angle Scattering Data from Colloids and Polymer Solutions: Modeling and Least-Squares Fitting. *Advances in Colloid and Interface Science* **1997**, *70*, 171–210. https://doi.org/10.1016/S0001-8686(97)00312-6.
(5) Bevington, P. R. *Data Reduction and Error Analysis for the Physical Sciences*; McGraw-Hill, 1969.
